# Physical Exercise and Immune System in the Elderly: Implications and Importance in COVID-19 Pandemic Period

**DOI:** 10.3389/fpsyg.2020.593903

**Published:** 2020-11-19

**Authors:** Fabiana Rodrigues Scartoni, Leandro de Oliveira Sant’Ana, Eric Murillo-Rodriguez, Tetsuya Yamamoto, Claudio Imperatori, Henning Budde, Jeferson Macedo Vianna, Sergio Machado

**Affiliations:** ^1^Department of Physical Education, Catholic University of Petrópolis, Petrópolis, Brazil; ^2^Sport and Exercise Sciences Laboratory, Catholic University of Petrópolis, Petrópolis, Brazil; ^3^Postgraduate Program in Physical Education, Federal University of Juiz de Fora, Juiz de Fora, Brazil; ^4^Molecular and Integrative Neuroscience Laboratory, Escuela de Medicina, División Ciencias de la Salud, Universidad Anáhuac Mayab, Mérida, Mexico; ^5^Intercontinental Neuroscience Research Group, Mérida, México; ^6^Graduate School of Technology, Industrial and Social Sciences, Tokushima University, Tokushima, Japan; ^7^Department of Human Sciences, European University of Rome, Rome, Italy; ^8^MSH Medical School Hamburg, Hamburg, Germany; ^9^Laboratory of Physical Activity Neuroscience, Physical Activity Sciences Postgraduate Program, Salgado de Oliveira University, São Gonçalo, Brazil; ^10^Laboratory of Physical Activity Neuroscience, Neurodiversity Institute, Queimados, Brazil

**Keywords:** COVID-19, elderly, health promotion, immune system, physical exercise

## Abstract

Physical exercise is seen as the main ally for health promotion, preventing and protecting the organism from several diseases. According to WHO, there is a tendency of constant growth in the elderly population in the coming years. The regular practice of exercises by the elderly becomes relevant to minimize the deleterious effects of the aging process and to increase the fitness index. Recently, the world population started a confrontation against Corona Virus Disease (COVID-19), which is the most significant public health challenge globally. Although social isolation is a reasonable measure in an attempt to stop contamination by COVID-19, this measure has limited the ability of individuals to exercise outdoors or in gyms and health clubs, which increased the risk of developing chronic illnesses related to a sedentary lifestyle. The critical point is that the recent recommendations on exercise prescription to combat the potentially harmful effects of COVID-19 failure to adequately address resistance exercise interventions as home-based exercise strategy. Thus, in this paper, we discussed the physical exercise as medicine if the training status is enough to protect the elderly against COVID-19 infection, about the role of physical activity on immunosuppression. Possible risks for COVID-19 infection, and the old training methods, such as no-load resistance training as possible resistance exercise strategies and high-intensity interval training, as new proposals of home-based exercise interventions, could perform during the current COVID-19 pandemic.

## Introduction

Physical exercise is seen as the main ally for health promotion, preventing and protecting the organism from several diseases (Garber et al., 2011). According to World Health Organization (2018), there is a tendency of constant growth in the elderly population in the coming year. Additionally, it is well-established that the practice of physical exercise is essential for well-being in the elderly population (Fletcher et al., 2018; Lavie et al., 2019; Sant’Ana et al., 2020).

Recently, the world population started the confrontation against (COVID-19), which today is the most significant public health challenge in the world (Park, 2020). Its high transmission capacity, even during the asymptomatic phase and the relatively low virulence, resulted in the rapid transmission of the virus in several continents, giving it a prominent role mainly due to its capacity for easy transmission through the airways and mucous membranes, and its significant lethality (Liu et al., 2020; Müller et al., 2020), in addition to the high mortality rate worldwide (Huang et al., 2020).

Determined as a pandemic (when on a large scale of severity in most parts of the world), organizations responsible for the prevention, maintenance, and treatment standards related to human health outlined some measures: social distancing (Team, 2020; WHO, 2020). Since then, this has been the primary strategy in the fight against COVID-19 in several countries in the world (Gasmi et al., 2020). As a result, the limited practice of physical exercise became a significant concern for the elderly population.

According to the WHO (2020), the elderly are considered a risk of contagion from COVID-19 due to vulnerability and death due to physiological fragility caused by the aging process. And physical exercise in this situation becomes an essential tool for the efficiency of immune function during aging (Bartlett et al., 2018; Schroeder et al., 2019).

The main cardiovascular changes associated with aging occur in the myocardium, in the sinoatrial node, and in the heart valves and blood vessels, characterizing both anatomical and functional changes (Affiune, 2002; Liberman, 2005).

The respiratory system undergoes a series of morphological and physiological changes, such as the accentuation of dorsal kyphosis, an increase in the anteroposterior diameter of the chest, a decrease in wall mobility, and atrophy of the respiratory muscles (Powers and Howley, 2015). There is a progressive loss of lung function (Ruivo et al., 2009), which is related to the loss of muscle mass manifested systemically, as well as the respiratory system, which occurs associated with a decrease in mobility and of thoracic compliance, with efficiency and pulmonary function (Salicio and Botelho, 2018).

However, physical exercise is a powerful ally for improving health (Schroeder et al., 2019), as it acts efficiently on the elderly immune function. It was reflected in better systemic functioning (Walsh and Oliver, 2016), mainly preventing infectious diseases (Walsh et al., 2011). Thus, in sedentary individuals, the capacity of the immune system is more deficient (Gleeson, 2004), but individuals who go into detraining have significant physiological losses (Reis et al., 2017; Blocquiaux et al., 2020), mainly in the immune function (Ferreira et al., 2020) increasing immunoprotective deficiencies.

Physical exercise is recommended in this pandemic period, which is complicated, mainly due to the high spread index of COVID-19 (Oliveira et al., 2020; Amatriain-Fernandez et al., 2020a,b); however, there is still no consensus or recommendations in the literature on this theme for the elderly population. Thus, in this opinion, we approach the importance of physical exercise in aging to maintain or improve the immune system, to protect against possible infections such as COVID-19, as well as to discuss the potential use of some methods of neuromuscular and cardiopulmonary training, as possible interventions that can be performed during the current pandemic period.

## Exercise is Medicine for Health Promotion

The act of staying at home without performing activities of daily living can involuntarily increase sedentary behavior and decrease the level of physical activity, which can cause negative health consequences. The reduction in levels of physical activity will decrease mechanical load, metabolic rate, and energy expenditure, resulting in decreased physical conditioning and excess energy. All of these risk factors are well-documented in the literature as they are the cause of several diseases, leading to a higher economic burden on society in the near future, besides, to be a severe public health problem (Owen et al., 2010; Malm et al., 2019).

It is well-known that a large part of the world population is very far from the minimum levels of physical exercise (Garber et al., 2011) recommended by institutions responsible for organizing and filing information related to the practice of physical activities, such as the ACSM (Katzmarzyk et al., 2019). However, due to the forced isolation and hypokinetic behavior experienced due to quarantine, a cascade of disastrous events can occur, such as respiratory tract infections (Hall et al., 2020).

A high level of physical inactivities, such as sitting for a long time, and low levels of physical activity are closely associated with increased risk of developing diseases such as depression (Huang et al., 2020), cancer and type 2 diabetes (Patterson et al., 2018), and coronary vascular disease and mortality (Stamatakis et al., 2019). For example, a 2-week reduction in physical activity levels causes a decrease in cardiorespiratory fitness (Bowden Davies, 2018) and a week of sedentary behavior, which leads to impairment in mood and depression (Edwards and Loprinzi, 2016). Also, sedentary behavior, for example, screen time, is a vital risk factor for venous thromboembolism (Kubota et al., 2018). However, the regular practice of physical activity and decreased levels of sedentarism are associated with reduced risk for morbidity and mortality (Ekelund et al., 2019).

Covid-19 causes several garve effects, such as lung damage, pneumonia (Liu et al., 2020), abnormal coagulation characteristics (Tang et al., 2020), and heart and kidney damage (Chen et al., 2020). Thus, prevention on the part of physical activity is of great interest. In this sense, some points are important. First point, the regular practice of physical activity promotes the improvement of cardiovascular functioning (Pinckard et al., 2019), coagulation and fibrinolytic homeostasis (Lippi and Maffulli, 2009), and protective effects against cellular stress (Narasimhan and Rajasekaran, 2016). Second point, the practice of physical activity increases cardiorespiratory conditioning (McKenzie et al., 2012), as well as the immune system (Dorneles et al., 2020), acting against the immunosenescence generally observed during aging (Weyh et al., 2020) and thus, increasing the immune response to viral antigens, reducing the incidence of viral infections throughout life (Campbell and Turner, 2018).

Physical activity during aging acts by promoting the activity of the immune system, including T and B lymphocytes (Bertucci et al., 2016). An example would be the release of interleukin-15 (IL-15) in muscle fibers after exercise, a crucial protein for the activation and proliferation of T and natural killer (NK) cells (Nielsen and Pedersen, 2007). This release induced by physical exercise can act in favor of the immune system, due to the many protective effects caused by exercise, such as the prevention of mild inflammation generally observed in central obesity (Collao et al., 2020), with a strong association with diabetes type 2 and coronary vascular disease.

And finally, the practice of physical activity also plays an essential role in mental health and cognitive functioning, since exercise promotes positive effects in the prevention and relief of symptoms of depression (Schuch et al., 2016), in reducing symptoms of anxiety (Stubbs et al., 2017), in improving learning (Winter et al., 2007), in addition to improving the cognitive functioning of the elderly (Bangsbo et al., 2019). In addition, the practice of physical activity and sports provides the practitioner with greater social interaction, important for the development of a social network (Holt et al., 2017). With limited social activities due to mandatory restrictions, physical and sports activities will remain considerably reduced during the outbreak of the virus. Therefore, the practice of regular physical activity is invaluable to maintain good physical and mental health when facing the current challenges imposed by COVID-19.

## Is the Training Status Enough as Protection Against COVID-19 Infection?

COVID-19, according to Carda et al. (2020), has different clinical manifestations that present themselves as mild, moderate, and severe. The most observed are without dyspnea and low oxygen saturation in the blood (SatO_2_), with or without feverish spikes, loss of smell and taste; dyspnea at small and medium efforts SatO_2_ 94–98% and radiological signs of pneumonia; dyspnea, SatO_2_ ≤93%, with the respiratory rate (RR) > 30/min, a radiological progression of the lesions, need for O_2_ supplementation, possibly with non-invasive ventilation; and finally, patients require mechanical ventilation, respectively.

It is already well-known that the practice of physical exercises acts as a positive measure in improving the individual’s chronic immune system (Brolinson and Elliott, 2007; Ranieri et al., 2009; Nieman and Wentz, 2019; Peake, 2020). In the general population, many protective immune responses are impaired in old age, leading to an increased risk of infection (Venkatraman and Fernandes, 1997). It is evident that a decline in the function of T cells occurs gradually, and this affects the whole immune system (Makinodan and Kay, 1980; Yan et al., 2001; Jeurissen et al., 2003; Ranieri et al., 2009; de Assis et al., 2018).

The immune system’s response to exercise is multifaceted, depending on its nature (Civinski et al., 2011; Nieman and Wentz, 2019). However, its regular and systematic practice acts in the prevention and complementary treatment for chronic diseases and viral infections such as the new coronavirus (Campbell and Turner, 2018; Ferreira et al., 2020; Halabchi et al., 2020; Wu et al., 2020; Zbinden-Foncea et al., 2020). It is becoming an indispensable factor in the protective effect of the immune system to respond to the threat of COVID-19 adequately.

In theory, exercise should help reverse the adverse effects of aging on the immune system, increasing the production of endocrine hormones that can contribute to less accumulation of autoreactive immune cells, improving programed cell death (Maughan et al., 2000; de Oliveira et al., 2010; Civinski et al., 2011). Besides, initial reports on COVID-19 show that elderly comorbidities are more likely to develop severe complications after COVID-19 infection and have an increased risk of mortality (Emami et al., 2020). These observations indirectly indicate that low fitness, obesity, and an altered immune system can be harmful to an individual exposed to the coronavirus, SARS-CoV-2. The effects of COVID-19 on patients with obesity have not been well-described; however, several reports have identified obesity as a risk factor for hospitalization (Dietz and Santos-Burgoa, 2020).

## Exercise, Immunosuppression, and Possible Risks for COVID-19 Infection

Starting a sudden state of confinement implies a change in the lifestyle of the population. These lifestyles and behaviors, in many cases, include a certain level of physical activity and exercise to maintain a good state of health (Jiménez-Pavón et al., 2020) to counteract the negative consequences of certain diseases (Sugiyama et al., 2008) or even to ensure active aging, reducing the risk of frailty, and illnesses associated with aging (Biswas et al., 2015; Fletcher et al., 2018).

Exercise is considered beneficial based on epidemiological evidence in disease prevention (Decoster et al., 2005; Kaminsky et al., 2019; Rognmo and Wisløff, 2019; Hall et al., 2020). In this sense, the saving older woman deserves special attention since physical activity and exercise have an impact on the harmful effects of Aging (Ranieri et al., 2009; Owen et al., 2010; ACSM, 2020) not being lifestyle change is convenient.

The main question that permeates in the health area regarding the practice of physical exercise is whether it is adequate and responds positively to the viral respiratory tract epidemic or not (Halabchi et al., 2020). In an integrative review on precautions and recommendations for the practice of physical exercise in the face of COVID-19, Nogueira et al. (2020) found that physical activity performed with moderate intensity has positive effects on the responses of the immune system against respiratory infections viral and is associated with several anti-influenza benefits, including reduced risk and increased rates of vaccine efficacy.

Miles (2009) stated that exercises with moderate intensity could increase the count of neutrophil cells, NK, and the increase in salivary IgA concentrations. In addition to increasing hormones of stress-reducing excessive inflammation and leading to increased immunity against viral infections by altering the responses of Th1/Th2 cells, influencing the immune response. Thus, the benefits of regular physical activity improve immune function, reduce the risk, duration, or severity of viral infections (ACSM, 2020; Hall et al., 2020).

## Old Training Methods as New Proposals of Home-Based Exercise Interventions

Regarding the regular practice of physical exercises, it is well-established that regular physical training is important for improving neuromuscular (Fragala et al., 2019), cardiovascular (Lavie et al., 2015) and cardiorespiratory capacities (Ferreira, 2017), cognitive function (Northey et al., 2018), and thus generating several other systemic improvements in older people (Nelson et al., 2007; Fletcher et al., 2018). Neuromuscular training enables more significant muscle responses to support the different functional capacities of this population (Fragala et al., 2019). Also, improvement of aerobic capacity is essential for the aging process (Molmen et al., 2012), enabling greater cardiovascular (Soares-Miranda et al., 2014), and cardiorespiratory functioning (Ferreira et al., 2012). Public health entities consider the elderly as a risk group during the COVID-19 pandemic. It is not recommended that they go to clubs, gyms, squares, and other locations to practice physical exercise, which can also aggravate (Mitchell et al., 2019).

However, in a period when social distance has recommended in all nations, the practice of physical exercises that do not require specific places and/or devices seems to be a positive and essential proposal in times of extreme health care, thus making it possible to maintain or gain physical conditioning (Nelson et al., 2007). However, physical exercises using body weight can be an efficient strategy for the elderly. Regarding the exercise intensity, the American College of Sports Medicine (ACSM) recommends moderate intensity with 150–300 min per week (ACSM, 2020). But it seems that high-intensity interval training (HIIT) with 75 min weekly, that is, short periods of length, can be a great strategy to achieve optimal levels of immunity in the elderly (Nieman et al., 1993; Nemoto et al., 2007; Adamson et al., 2019).

Using bodyweight exercises can also be an option of HIIT composed by sets of short stimuli (below 1 min) or few repetitions (up to 12 repetitions) but with higher execution speeds (according to motor capacity). However, following these guidelines, HIIT, with up to 10 min (total volume), can be an effective strategy for positive physiological adaptations in older people (Tjønna et al., 2018). Besides, HIIT of short volume does not seem to promote damage to the clinical status of elderly individuals, as soon as it does not seem to negatively affect the immune system (Taylor et al., 2019). The recovery time, to preserve the body from physiological stress, using intervals longer than the stimuli is more beneficial to the body (e.g., 1:2; 1:3; 1:4; Winett and Ogletree, 2019).

However, it is necessary to monitor the intensity during and after the training sessions mainly. Thus, the subjective perception of effort (SPE) is more indicated to be used during the practice of exercise, even for older people (Borg, 1982), as well as percentages of heart rate (HR; Tanaka and Melo, 2001), to achieve the better intensity control. In HIIT, monitoring by PSE can offer better responses (Laursen and Buchheit, 2019). Regarding the training monitoring, the use of the OMNI-RES scale can be an excellent tool to control intensity (Silva-Grigoletto et al., 2013). For the elderly, another factor that may offer inefficiency of training monitoring is the use of medications that interferes in cardiac behavior, such as beta-blockers, widespread in this population (Taylor et al., 2019).

Additionally, the practice of exercises to improve overall health condition, the control of training responses (internal load) is important to avoid possible stresses and negative effects on the immune system (Meeusen et al., 2013), mainly in elderly individuals (Nieman et al., 1993). Therefore, the method of monitoring internal load through SPE (Foster et al., 2001) is effective and can be an important ally in preventing metabolic stress, which directly interferes with the immune functions (Joisten et al., 2019). Another important method of monitoring the internal load is through the baseline HR, where constant changes (for more) of the baseline HR values can be considered probable stress and, thus, changes in training prescription must be done (Schneider et al., 2018).

## Conclusion

Staying physically active during social restriction is essential in promoting health and preventing future chronic conditions resulting from a sedentary lifestyle. In this period of social isolation, medical care and vital social services must be a priority. Thus, the prevention of physical and mental suffering should be a priority on the part of governments and public health authorities, encouraging the maintenance of physical activity during the COVID-19 pandemic.

Summarizing, all physical activity is beneficial, and any practice is better than doing nothing. It is also essential to reduce the sedentary lifestyle and accumulate at least 150 min of moderate-intensity physical activity or 75 min of vigorous-intensity physical activity per week is mandatory.

## Author Contributions

FS, LS, and SM developed the study concept and wrote the first draft of the manuscript, and the later drafts of the manuscript were adjusted by FS, LS, EM-R, TY, CI, HB, JV, and SM in collaboration. All authors contributed to the article and approved the submitted version.

### Conflict of Interest

The authors declare that the research was conducted in the absence of any commercial or financial relationships that could be construed as a potential conflict of interest.
